# Use of Interspecies Correlation Estimation (ICE) Models to Derive Water Quality Criteria of Microplastics for Protecting Aquatic Organisms

**DOI:** 10.3390/ijerph191610307

**Published:** 2022-08-19

**Authors:** Jiangyue Wu, Xiaohui Zhao, Lin Gao, Yan Li, Dan Wang

**Affiliations:** 1 National Marine Hazard Mitigation Service, Ministry of Natural Resource of the People’s Republic of China, Beijing 100194, China; 2 Department of Water Ecology and Environment, Institute of Water Resources and Hydropower Research, Beijing 100038, China

**Keywords:** microplastics (MPs), water quality criteria (WQC), interspecies correlation estimation (ICE), species sensitivity distribution (SSD)

## Abstract

Microplastics (MPs) in the water environment pose a potential threat to aquatic organisms. The Species Sensitivity Distribution (SSD) method was used to assess the ecological risks of microplastics on aquatic organisms in this study. However, the limited toxicity data of aquatic organisms made it impossible to derive water quality criteria (WQC) for MPs and difficult to implement an accurately ecological risk assessment. To solve the data gaps, the USEPA established the interspecies correlation estimation (ICE) model, which could predict toxicity data to a wider range of aquatic organisms and could also be utilized to develop SSD and HC_5_ (hazardous concentration, 5th percentile). Herein, we collected the acute toxicity data of 11 aquatic species from 10 families in 5 phyla to fit the metrical-based SSDs, meanwhile generating the ICE-based-SSDs using three surrogate species (*Oncorhynchus mykiss*, *Hyalella Azteca*, and *Daphnia magna*), and finally compared the above SSDs, as well as the corresponding HC_5_. The results showed that the measured HC_5_ for acute MPs toxicity data was 112.3 μg/L, and ICE-based HC_5_ was 167.2 μg/L, which indicated there were no significant differences between HC_5_ derived from measured acute and ICE-based predicted values thus the ICE model was verified as a valid approach for generating SSDs with limited toxicity data and deriving WQC for MPs.

## 1. Introduction

Significant evidence shows that Microplastics (MPs) have entered the water environment and threaten the well-being of aquatic species [1,2,3]. Microplastics are defined as plastic particles, fragments, fibers, and films up to 5 mm in size [4,5,6,7]. Previous studies have concluded that MPs had a variety of toxic effects on aquatic organisms, such as lethal toxicity, enzyme toxicity, genetic toxicity, thyroid toxicity, and reproductive toxicity, etc. [8,9,10]. Results showed that MPs might impose potential risks to the aquatic ecosystem, including oceans [11]. The bulletin of China’s marine ecological environment in 2021 showed that the average density of floating MPs on the sea surface was 0.44 pieces/m^3^. This pollution cannot be ignored [12]. The fundamental reason for the difficulty in conducting a comprehensive aquatic risk assessment for MPs is the lack of toxicity data [13].

Species Sensitivity Distribution (SSD) is an important instrument for developing Water Quality Criteria (WQC). However, due to the complexity of test protocols and species availability, results for certain species were unlikely to be obtained. In response to the data gap, the US EPA developed the interspecies correlation estimation (ICE) model, which is based on a log–log correlation between abundant chemical toxicity values for a series of species [14], at least 1258 chemicals, and 5487 test results of 180 species in an acute toxicity dataset [15]. Moreover, the ICE model could accurately predict the toxicity and produce protective toxicity estimates [16], such as species sensitivity distribution (SSD) and HC_5_ (hazard concentration, 5th percentile), for evaluating the contaminant threat to species of interest [17,18,19,20]. The ICE will help overcome the lack of toxicity data to ensure that there is enough data for WQC studies [9,21].

In this study, we collected the acute toxicity data of 11 aquatic species from 10 families in 5 phyla to fit the metrical-based SSDs, meanwhile generating the ICE-based-SSDs using three surrogate species (*Oncorhynchus mykiss*, *Hyalella Azteca*, and *Daphnia magna*), and finally compared the above SSDs, as well as the corresponding HC_5_. Moreover, the WQC of MPs for aquatic organisms was derived using a battery of toxicity data (11 aquatic species from 10 families in 5 phyla) according to the US EPA guidelines.

The objectives of this study were to (i) derive measure-based and ICE-based WQC for MPs and to (ii) compare measured-based-SSDs with original and improved ICE-based-SSDs for MPs. Our study could provide valuable information on pollution management and environmental risk assessment for MPs in the ambient aquatic environment.

## 2. Materials and Methods

### 2.1. Measured Toxicity Data Collection and Processing

ECOTOX (http://cfpub.epa.gov/ecotox, accessed on 1 March 2022) and two supplemental online databases, CNKI (http://www.cnki.net, accessed on 1 March 2022) and ELSEVIER (http://www.sciencedirect.com, accessed on 1 March 2022), were used to obtain MP toxicity data. The data were checked and processed in accordance with EPA requirements in the United States [22]. “MPs”, “Microplastics”, “aquatic life/organisms”, “toxicity”, and “ecotoxicity” were the important terms.

Data were submitted to stringent quality control procedures and subjected to rigorous quality assurance guidelines (Yan et al., 2013). To begin with, the values of 48-h LC_50_ or EC_50_ for *Daphnia* and 96-h LC_50_ or EC_50_ for other species were discovered in databases or literature for aquatic acute toxicity. Second, the data’s key toxicological endpoints were immobility, respiratory inhibition, and mortality. Finally, the vast majority of exposure tests were either flow-through or static/renewal. All of the tests were carried out in accordance with ASTM standards [21]. The detailed information about the Measured toxicity data is listed in Table 1. The acute toxicological data of 11 aquatic species from 10 families in 5 phyla were collected. The most sensitive species were *Tetraselmis chuii*, i.e., a kind of algae. As we all know, algae are one of the important components of marine primary productivity [23] and can take the lead in sensing microplastic pollution in water. The least sensitive species were *Vibrio fischeri*, the EC_50_ was 1.00 × 10^6^ μg/L.

### 2.2. ICE Data Set

The ICE software and more powerful ICE models for aquatic and terrestrial species were created by the US EPA and made accessible on the internet (https://www3.epa.gov/webice/, accessed on 1 March 2022). In this study, the Web-ICE platform was employed. Through a user-friendly interface, the Web-ICE delivered interspecies extrapolation models for acute toxicity. The following requirements were satisfied with the test results of the database: Fish with an LC_50_/EC_50_ of 96 h; most invertebrates with a LC_50_/EC_50_ of 48 h; fish weighing 0.1–0.2 g; fish less than 1 months old or less than 30 mm in length. Furthermore, MPs’ toxicological endpoints in fish and invertebrates are limited to those associated with death, such as immobility, respiratory suppression, and fatal impacts.

Based on geometric means from the measured database, the Web-ICE was seeded with acute toxicity values for *Oncorhynchus mykiss*, *Hyalella Azteca*, and *Daphnia magna* (6.03 × 10^5^ μg/L, 2.18 × 10^5^ μg/L, and 7.70 × 10^2^ μg/L, respectively) to predict toxicity values.

### 2.3. Data Analysis

Log-logistic, log-normal, and Burr III were usually used to fit SSDs [34]. Based on previous studies, we found that log-logistic fitted the toxicity data well and could make it statistically more meaningful. Thus, in this study, log-logistic was used to generate SSDs. The equation is shown as follows:

Y = 1/(1 + exp ((α − X) / β))(1)

where the cumulative probability of species is defined as Y, which is the order of the data point divided by one plus the total number plus one of the data points; X is the LC_50_ or EC_50_ by log-transformed. Where α and β are parameters, which represent the location (or intercept) and the slope of the curve, respectively. Moreover, we used a two-sample Kolmogorov–Smirnov test (K-S test) to analyze the difference between the predicted data group and the measured data group [35]. The main data analysis software was Origin 8.0 and SPSS 20.0.

According to the US EPA guidelines, the criteria used to pick the predicted toxicity data are briefly listed as follows: (1) mean square error (MSE) < 0.22; (2) taxonomic distance ≤ 4; (3) cross-validation success rate > 85%; (4) degree of freedom (df) > 8; (5) R^2^ value > 0.6; (6) *p*-values < 0.01. The statistical parameters were critical to assessing the accuracy of the model [34,35,36].

## 3. Results and Discussion

### 3.1. Estimated Toxicity Using Web-ICE

By running the Web-ICE program, we obtained 133 predicted toxicity values, and only 19 toxicity data were adopted based on the aforementioned ICE criteria, including amphibians, invertebrates, and fish (see Table 2). One hundred and fourteen toxicity data did not meet one or more conditions, such as MSE > 0.22 or R^2^ value ≤ 0.6, or cross-validation success rate ≤ 85%.

Fifty-one toxicity values of different species were predicted by Web-ICE for D. magna. Forty-two invalid data were excluded, and only seven species were effective. They were Thamnocephalus platyurus, Daphnia pulex, Simocephalus serrulatus, Utterbackia imbecillis, Amblema plicata, Megalonaias nervosa, and Margaritifera falcata. For O. mykiss, 9 effective data came from a total of 62 data. For H. azteca, three effective data came from a total of 20 data.

### 3.2. ICE-and Measure-Based SSD

**Figure 1:**
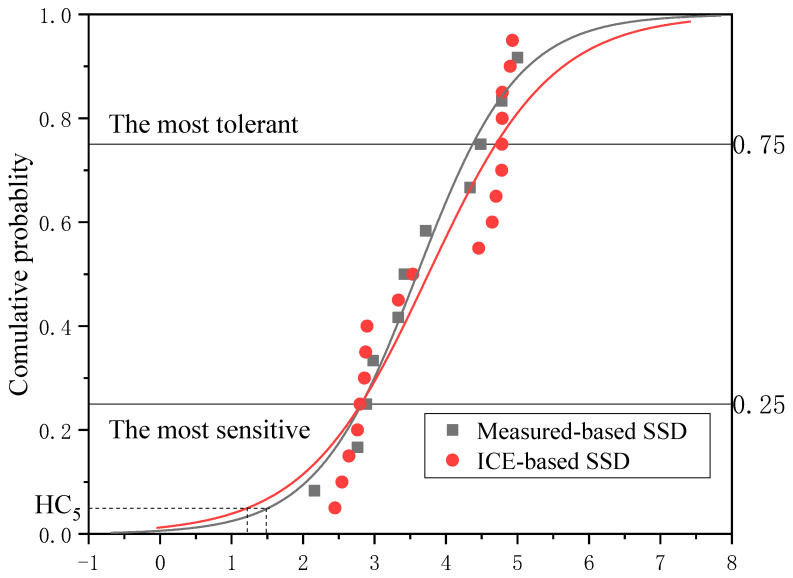
Comparison of SSDs constructed using measured toxicity data and ICE predicted toxicity data from 3 surrogate species.

SSDs were generated by log-logistic, which was constructed using measured toxicity data and ICE predicted toxicity data from three surrogate species (Figure 1). The cumulative probability means the sensitivity of species. The results showed that species in the first quartile of the SSD curve were assumed to be the most sensitive, while those in the second and third quartiles were supposed to be moderately tolerant, and those in the fourth quartile were thought to be the most tolerant [36,37,38]. The ranking results of the predicted and the metrical species distributions were quite different, the most sensitive species based on the metrical SSD curves were *Tetraselmis chuii*, *Pseudokirchneriella subcapitata*, and *Daphnia magna* (a total of 11, a quartile of 3), while the most sensitive species in ICE-based SSD curves were *Amblema plicata*, *Americamysis bahia*, *Megalonaias nervosa*, *Ceriodaphnia dubia*, *Utterbackia imbecillis*, and *Daphnia pulex* (a total of 19, a quartile of 5). The most tolerant species were a little different between the two ICE models. The most tolerant species predicted by original ICE models were *Salmo trutta*, *Oncorhynchus tshawytscha*, and *Oncorhynchus kisutch* (a total of 12, a quartile of 3), while the most sensitive species predicted by improved ICE models were *Oncorhynchus tshawytscha*, *Ictalurus punctatus*, *Oncorhynchus kisutch*, *Pimephales promelas*, and *Carassius auratus* (a total of 21, a quartile of 5). Dyer et al. [19] used ICE and ranked the predicted species. The result suggested that those more sensitive to a wide range of chemicals were cold-water fish species Moreover, Raimondo et al. [38] also concluded that trout was the most sensitive species to pesticides via ICE predicted values. That is not consistent with this study, the sensitivity of various species to specific chemicals was also observed to be significantly different [37].

In addition, the most sensitive species and the most tolerant species were both predicted by improved ICE models, which showed a much wider range of predictions than the original ICE models [34,35,36,37]. Although HC_5_ values obtained from ICE- and measure-based SSD were different, the Kolmogorov–Smirnov test indicated that the two SSDs showed no significant difference (ks = 0.902, n1 = 11, n2 = 19, *p* = 0.183 > 0.05).

### 3.3. Aquatic Life Criteria Derivation

In this study, the HC_5_ of MPs from ICE- and measure-based SSD were obtained to be 167.2 μg/L and 112.3 μg/L, respectively, which were similar to those reported in previous studies [39,40]. The comparison results suggested the possibility of extrapolation utilizing ICE in terms of statistical analysis and effect evaluation. As a result, the use of ICE models to produce relatively accurate estimates of chemical toxicity and protective criteria was encouraging, and it might be utilized as a potential alternative to current water quality derivation techniques in the absence of appropriate empirical toxicity data [41,42,43].

While there are solid ecological reasons to avoid using non-China specifics in the development of China-specific water quality criteria, considerable new research on the evaluation of ecological features as elements worth protecting may lead to reconsideration of such eliminations. If attributes are protected, the location of the species endemicity is irrelevant. This is crucial to assessing danger at a given location. We envisage the creation and deployment of ICE models that include a wide range of species from throughout the world, providing the most rigorous technique for developing environmental criteria regardless of location.

## 4. Conclusions

This study compared HC_5_ obtained from ICE-based and metrical acute toxicity values of MPs for aquatic species to assess the accuracy of ICE-generated SSDs. There was no significant difference between the ICE-based and measured-based SSDs, showing that ICE might be a viable method for predicting acute toxicity data for aromatic chemicals. Furthermore, the collection of toxicity data from experiments usually suffered from a long cycle and high cost. Compared with the experimental measurement, the cost of the prediction via ICE models is fairly low, which would save significant time and expense. Thus, in ecological risk assessment, the ICE models are advocated as a good option.

## Figures and Tables

**Table 1:** Toxicity data of microplastic to aquatic species (LC_50_/EC_50_).

Phylum	Family	Species	LC_50_/EC_50_ (μg/L)	Reference
Arthropoda	Daphnidae	*Daphnia magna*	7.70 × 10^2^	[23]
		*Ceriodaphnia dubia*	9.58 × 10^2^	[24]
	Thamnocephalidae	*Thamnocephalus platyurus*	5.20 × 10^3^	[25]
	Harpacticidae	*Tigriopus japonicus*	2.15 × 10^3^	[26]
	Hyalellidae	*Hyalella azteca*	2.18 × 10^5^	[27]
Chordata	Salmonidae	*Oncorhynchus mykiss*	6.03 × 10^5^	[28]
	Gobiidae	*Pomatoschistus microps*	3.05 × 10^5^	[29]
Chlorophyta	Chlorodendraceae	*Pseudokirchneriella subcapitata*	5.80 × 10^2^	[30]
		*Tetraselmis chuii*	1.45 × 10^2^	[31]
Proteobacteria	Vibrionaceae	*Vibrio fischeri*	1.00 × 10^6^	[32]
Echinodermata	Parechinidae	*Paracentrotus lividus*	2.61 × 10^3^	[33]

**Table 2:** Summary of the regression parameters of surrogate-predicted species using ICE models.

Surrogate Species	Predicted Species	Estimated Toxicity (mg/L)	Cross-Validation Success (%)	MSE	R^2^	Taxonomic Distance
***Daphnia magna***						
	*Thamnocephalus platyurus*	724.26	91	0.05	0.98	4
	*Daphnia pulex*	628.65	90	0.12	0.97	1
	*Simocephalus serrulatus*	755.41	87	0.21	0.88	2
	*Utterbackia imbecillis*	580.28	100	0.11	0.96	4
	*Amblema plicata*	279.27	90	0.18	0.94	4
	*Megalonaias nervosa*	437.8	91	0.16	0.96	3
	*Margaritifera falcata*	787.86	90	0.14	0.95	3
***Oncorhynchus mykiss***						
	*Salmo salar*	61,347.21	93	0.12	0.95	2
	*Salvelinus fontinalis*	60,703.33	92	0.11	0.94	2
	*Salmo trutta*	61,269.36	96	0.1	0.95	2
	*Oncorhynchus tshawytscha*	60,424.03	94	0.07	0.96	1
	*Oncorhynchus kisutch*	79,193.94	100	0.04	0.98	1
	*Oncorhynchus clarkii*	44,376.05	95	0.09	0.94	1
	*Lepomis cyanellus*	85,160.74	100	0.13	0.94	4
	*Salvelinus namaycush*	28,786.62	96	0.08	0.93	2
	*Perca flavescens*	50,142.3	88	0.14	0.94	4
***Hyalella azteca***						
	*Gammarus pseudolimnaeus*	2161.17	100	0.03	0.99	3
	*Pimephales promelas*	3457.14	97	0.22	0.85	4
	*Americamysis bahia*	350.71	86	0.20	0.86	4

## Data Availability

Data are available on request due to privacy and ethical restrictions.
